# Scale-free resilience of real traffic jams

**DOI:** 10.1073/pnas.1814982116

**Published:** 2019-04-12

**Authors:** Limiao Zhang, Guanwen Zeng, Daqing Li, Hai-Jun Huang, H. Eugene Stanley, Shlomo Havlin

**Affiliations:** ^a^School of Reliability and Systems Engineering, Beihang University, Beijing 100191, China;; ^b^National Key Laboratory of Science and Technology on Reliability and Environmental Engineering, Beijing 100191, China;; ^c^School of Economics and Management, Beihang University, Beijing 100191, China;; ^d^Center for Polymer Studies, Boston University, Boston, MA 02215;; ^e^Physics Department, Boston University, Boston, MA 02215;; ^f^Department of Physics, Bar-Ilan University, Ramat-Gan 52900, Israel

**Keywords:** resilience, scaling laws, spatiotemporal, traffic congestion, complex systems

## Abstract

The concept of resilience can be realized in natural and engineering systems, representing the ability of a system to adapt and recover from various disturbances. Although resilience is a critical property needed for understanding and managing the risks and collapses of transportation systems, an accepted and useful definition of resilience for urban traffic as well as its statistical property under perturbations are still missing. Here, we define city traffic resilience based on the spatiotemporal clusters of congestion in real traffic and find that the resilience follows a scale-free distribution in 2D city road networks and 1D highways with different exponents but similar exponents on different days and in different cities. The traffic resilience is also revealed to have a scaling relation between the cluster size of the spatiotemporal jam and its recovery duration independent of microscopic details. Our findings of universal traffic resilience can provide an indication toward better understanding and designing of these complex engineering systems under internal and external disturbances.

## Significance

Traffic congestion has become the most stubborn disease for the health of a city. Like the self-healing ability of a biological unit from diseases, transportation can also recover spontaneously from various disturbances. To describe this recovery, we define the resilience metric as the spatiotemporal congestion cluster, which can be used for other network systems. Based on large-scale GPS datasets, we reveal that the recovery behavior of transportation from congestion is governed by three scaling laws for all of the congestion scales. These scaling laws are found independent of microscopic details, including fluctuation of traffic demand and corresponding management. Our results of resilience scaling can help to better characterize and improve the adaptation and recovery of city traffic from various perturbations.

Increasing traffic congestion is an inescapable problem due to enhanced urbanization and growing metropolitan cities all over the world from Los Angeles to Tokyo and from Cairo to Beijing (1), leading to potential high economic and social losses. Under various internal or external perturbations ranging from a local flow fluctuation to a broken-down traffic light and up to extreme weather conditions, a small jam can develop into large-scale congestion in a domino-like cascading process (2). Given the uncertainty of disruptive system failures, the concept of resilience describes the system ability to withstand possible perturbations and recover to an acceptable functional level. Since Holling’s definition in ecology (3), the resilience framework has been developed and applied in many disciplines ranging from climate and economics to social science (4–12). System resilience across different domains usually depends on its absorptive capacity, adaptive capacity, and restorative capacity (13). Accordingly, system adaptation and recovery process in various critical infrastructures, including transportation, have attracted much attention recently (14–17). Specifically, a resilient transportation system in the future smart city era could improve life quality and the development of economic society significantly and reduce environmental pollution (18).

Transportation systems with network topology, as one of the critical infrastructures, serve as the lifeline for national economics and stability. System resilience has been studied in different traffic systems, including city roads, metro systems, freight transportation, and aviation networks (19–25). While different methods have been proposed to evaluate and improve the resilience of transportation and other infrastructures, the resilience metric is mainly based on a dimensionless indicator. Chang and Shinozuka (26) introduced this resilience measurement that relates expected losses in future disasters to a community seismic performance objective. It has been proposed to define for earthquakes the measurement of resilience as the change in system performance over time (27), which is the well-known resilience triangle. It measures the resilience loss of a community due to an earthquake using

$$RL=\int_{t_{0}}^{t_{1}}\left[1-Q\left(t\right)\right]dt.$$ [1]

Here, *Q*(*t*) represents the service quality (ranging between 0 and 100%) of the community, which starts to decrease at $t_{0}$ and may return to its normal state (100%) at $t_{1}$. Although this method is presented in the context of earthquakes, the concept has been widely applied to other scenario-specific system performance under various disturbances (28, 29). Meanwhile, although critical for understanding and improving the system robustness and vulnerability (30–37), the network topology has rarely been considered in resilience studies of critical infrastructures and other complex systems. Since the traffic system in a city has a typical network structure and its resilience evolves both in space and time, the above-mentioned dimensionless resilience indicators and relevant studies may have missed the spatiotemporal properties of system adaptation and recovery in these critical infrastructure networks.

Composed of a very large number of strongly interacting subunits, transportation systems are usually running out of equilibrium states with unpredictable outcome of cascading failures (38). Due to the longstanding debate of whether system resilience is intrinsic (39), it is critical yet unknown if such systems with numerous interacting subunits have universal resilient behavior that is independent of microscopic details. Here, we propose a spatiotemporal resilience measure, incorporating both spatial and temporal features of system adaptation and recover, to explore the possible universality features of traffic resilience. With extensive real traffic data, we find scaling laws from scale-free distributions for the traffic resilience and recovery duration. Our definition and results demonstrate and support the existence of intrinsic behavior behind traffic resilience independent of microscopic details. These scaling laws hold for different size scales of traffic-jammed clusters, which can help to predict system restoration behaviors and develop corresponding resilience management methods.

## Results

**Fig. 1.**
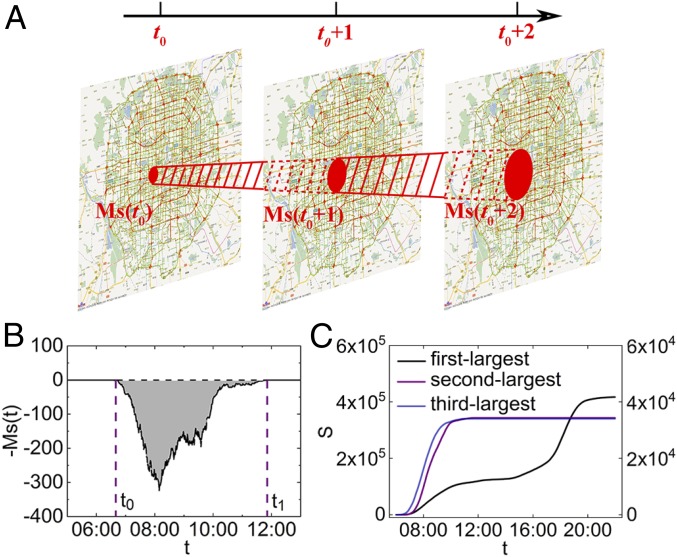
Traffic resilience defined based on spatiotemporal jammed clusters. (*A*) Illustration of the evolution of a jammed cluster in a city. Red links are considered congested. All red links in the shadow belong to the same jammed cluster. (*B*) The cross-section area $M_{s}\left(t\right)$ of the second largest jammed cluster on October 26, 2015 in Beijing. Since the resilience is reduced during the jam, we plot the negative of $M_{s}\left(t\right)$ as a function of time, and traffic resilience can be represented by the gray area. The gray area is the size of the spatiotemporal jammed cluster (*S*) shown in red in *A*. The timespan between $t_{0}$ and $t_{1}$ represents its recovery time (*T* = $t_{1}$ − $t_{0}$ + 1). (*C*) The cluster sizes of the first, second, and third largest jammed clusters on October 26, 2015 in Beijing as a function of time (the second and third largest clusters sizes are given on the right-axis scale).

Our study uses real traffic GPS data from Beijing and Shenzhen, which are two of the megacities that suffer from the most severe traffic jams worldwide and particularly, in China. Complex road topology, large traffic flow, and various perturbations as well as the availability of big data make these two megacities ideal for urban traffic research of resilience. The static road network in Beijing contains over 39,000 road segments (links) and 27,000 intersections (nodes), while the Shenzhen traffic network contains about 18,000 road segments (links) and 12,000 intersections (nodes). The dataset covers GPS velocity records in both cities for 30 d during October 2015 with resolution of 1 min. A dynamical traffic network can be constructed based on road topology information and high resolution of evolving traffic velocity data. Each road in the network has a velocity $v_{i}$ (kilometers per hour), and a given velocity threshold $p_{i}$ is determined to judge the traffic availability of this road (detailed thresholds for different roads are shown in *SI Appendix*, Table S1). We also tested the influence of the threshold and find that our results are insensitive to the thresholds (details are in *SI Appendix*, Figs. S1–S3). Then, roads with real-time velocity $v_{i}$ below the threshold are regarded as congested. Specifically, the links in the jammed cluster at a given time represent congestion roads, while nodes in the jammed cluster are the intersections between these congested roads. Considering together the temporal evolution as well as the 2D spatial traffic network, we can regard the jam as a 3D spatiotemporal network cluster. Accordingly, a 3D (two of space and one of time) cluster can be constructed to represent the same jam during its entire lifetime. The 3D jammed cluster is demonstrated in Fig. 1*A*, where all red links in the shadow belong to the same jammed cluster. Note that the connected clusters here do not necessary mean that any roads within a connected cluster are spatially connected at a given time instant. When a jammed cluster splits into two or more subclusters at a certain instant, all links and nodes in the subclusters still belong to the same 3D cluster due to their temporal connection. Our definition of jammed clusters intuitively reflects the spatiotemporal propagation and dissolution of traffic jams instead of earlier dimensionless resilience indicators.

We define the resilience based on the 3D cluster size using conceptually Eq. **2** as follows. For each jammed cluster during the observed period (e.g., from 0600 to 2200 hours), the number of its links (roads) at a snapshot of the temporal layer *t*, $M_{s}\left(t\right)$, varies with time. Thus, $M_{s}\left(t\right)$ can be regarded as the cross-section area of the jammed cluster at time *t*. Larger $M_{s}\left(t\right)$ means that more roads are congested at snapshot *t*. The maximal cross-section of the spatiotemporal congestion cluster in Beijing is also plotted (*SI Appendix*, Fig. S4). We evaluate the resilience performance of the traffic network by analyzing the evolution and statistics of $M_{s}\left(t\right)$. For example, the time evolution of $M_{s}\left(t\right)$ of the second largest jammed cluster on October 26, 2015 is demonstrated in Fig. 1*B*. The timespan between $t_{0}$ and $t_{1}$, which is the lifetime of this jammed cluster, is defined as the recovery duration (*T* = $t_{1}$ − $t_{0}$ + 1). The recovery duration reflects how long it takes for this jammed cluster to recover from the beginning of congestion. We define the cluster size *S* as the total number of links (roads) in the jammed cluster during its recovery time as

$$S=\int_{t_{0}}^{t_{1}}M_{s}\left(t\right)dt.$$ [2]

The cluster sizes of the first three largest jammed clusters on October 26, 2015 in Beijing as a function of time are demonstrated in Fig. 1*C*. The cluster size naturally represents the loss of resilience in the traffic network. Eq. **2** not only characterizes the propagation of congestion in spatial dimension but also, includes the duration of congestion. Thus, the larger the jammed cluster size is, the less resilient the traffic system should be regarded. The shadow area shown in Fig. 1*B* represents, therefore, this loss of traffic resilience. To show the daily variations in the cluster sizes, we plot the size of the first three largest clusters as a function of date in Beijing (*SI Appendix*, Fig. S5). The largest cluster sizes are found to be obviously smaller on holidays (October 1 to October 7, 2015) due to less traffic demand compared with normal workdays. Note that, when two (or more) jammed clusters merge, they will be regarded as a single 3D cluster. We update the information of all links and nodes in the subclusters and identify them as a single jammed cluster.

**Fig. 2.**
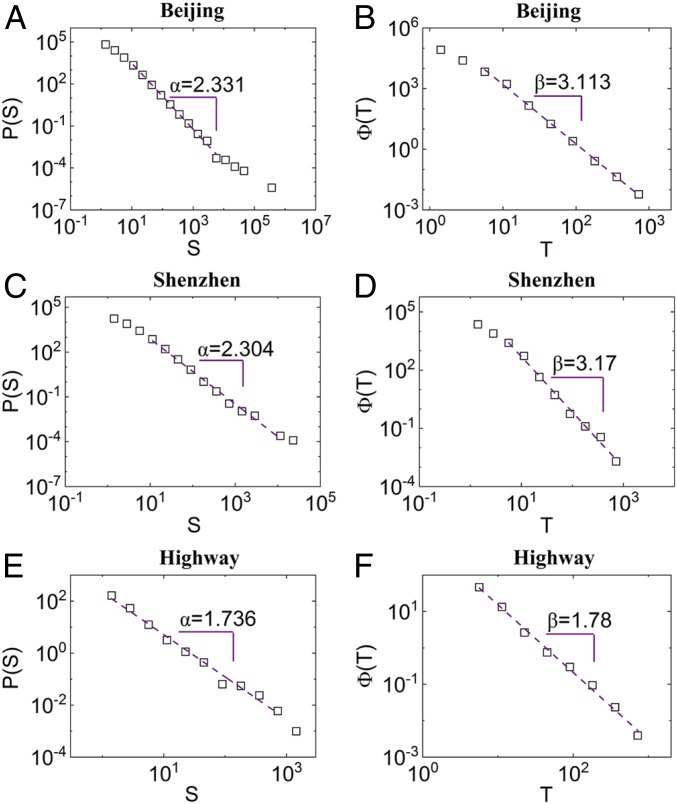
Scale-free distributions of traffic resilience. (*A*) The distribution of the jammed cluster size. (*B*) The distribution of recovery duration. *A* and *B* show typical results based on city traffic data in Beijing on October 26, 2015. *C* and *D* show typical results based on city traffic data in Shenzhen on October 26, 2015. *E* and *F* show typical results based on traffic data on the Beijing–Shenyang Highway on October 1, 2015. The results are analyzed by logarithmic bins and plotted in double-logarithmic axis.

Next, we explore the distributions of cluster sizes and recovery durations in a typical day. The results on Monday, October 26, 2015 in Beijing and Shenzhen are shown in Fig. 2. The distribution of cluster sizes shows a scale-free property (i.e., a power law scaling),

$$P\left(S\right)∼S^{-\alpha },$$ [3]

with an exponent α close to 2.3 in both cities. The power law distribution of cluster size suggests that, although most of the congestion is of small scale, there exist everyday congestions of sizes at all of the scales, including extremely large spatiotemporal scale. With signal control-based traffic management, small jams due to fluctuating traffic demand or accidents in a city will usually shrink and dissolve after a short time span. However, if the traffic supply under real-time management cannot meet the increasing traffic demand, the traffic jam will grow to a large scale and take more time to recover. These two behaviors compete in different scales in the city and possibly lead to the scale-free distribution of traffic resilience. This also suggests that, under different levels of internal or external perturbation, transportation systems have the same response distribution described by one scaling function.

**Fig. 3.**
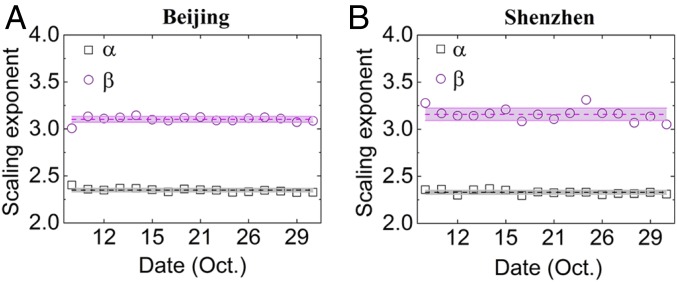
Scaling exponents of the scale-free distributions of cluster size and recovery duration as a function of date in (*A*) Beijing and (*B*) Shenzhen.

We also find that the cluster size distribution in both cities follows a very similar power law (α = 2.34 ± 0.02) for all observed workdays (Fig. 3). The high-quality scaling laws found here in different cities and different periods highly suggest that the resilience defined here may reflect an intrinsic property of urban traffic independent of the microscopic traffic details that change from day to day and from city to city. Since all sizes seem to follow the same scaling law, a unified resilience management may exist for different sizes and locations of jams.

Next, we analyze and explore the scaling properties of the recovery duration in traffic congestion. In Fig. 2*B*, we show the distribution on a typical day in Beijing. It is found that the recovery duration of jammed clusters follows a distinct power law distribution,

$$\Phi \left(T\right)∼T^{-\beta },$$ [4]

with an exponent β. Furthermore, similar results for the scaling exponent β are also found for another city: Shenzhen (Fig. 2*D*). In these two megacities, the power law distributions for system recovery are similar in all of observed days with β = 3.13 ± 0.06 (Fig. 3). Under different possible perturbations, there seems to exist all scales of recovery duration, including some cases of very long recovery duration, but all (short, medium, and long recovery durations) follow the same scaling law. This scaling law enables us to understand the common recovery mechanism for different sizes of jammed clusters, which would be helpful for mitigation guidance.

Surprisingly, the power law exponents of resilience cluster size and recovery duration distributions are found to be stable on different days in two cities during the observed period (Fig. 3). The appearance of the power law and its stability on different working days for a city are probably due to the self-organized nature (40) of traffic flow and corresponding optimized management in urban traffic. On the one hand, a large number of vehicles rush onto the road network during peak hours, which fluctuates from day to day. After the traffic flow returns to normal status, congestions disappear spontaneously. On the other hand, corresponding traffic control strategies, such as traffic diversion, traffic lights, and speed limitation, are applied to alleviate specific traffic jams on a given day and pursue the system efficiency (41, 42). All of these push the system toward its intrinsic operational limits, which might contribute to our findings of robust scale-free distributions of cluster sizes and recovery duration. Since our transportation system of a city is a large system with relatively similar daily flow demand and a corresponding traffic control strategy, this is similar to the sandpile model for self-organized criticality (40), where the critical state is also robust under perturbation. Our definition of spatiotemporal resilience in some sense measures the spatiotemporal-scale range of the attraction basin.

Our traffic system can be seen as analogous to the sandpile model, since “particles” (cars) are continuously added into the transportation system in a city starting early every morning. Then, a local perturbation of a traffic jam may develop and spread to neighboring sites, like a domino effect, forming congestion of all sizes as a result of the self-organized criticality similar to the sandpile model. This self-organized behavior generates spatial self-similar structures and temporal correlations across a broad range of scales, similar to the sandpile model. Here, we found that the spatial and temporal scaling behaviors interact and form a scale-free size distribution in the *d* + 1 resilience clusters. The universal features suggested by the scale-free nature of traffic resilience usually depend on a few macroscopic variables, including network dimension (43) and total traffic demand. To test this hypothesis, we also analyzed the traffic data of the Beijing–Shenyang Highway between October 1 and October 7, 2015. This observed time span is the National Day holiday in China, during which the highway is usually under heavy traffic pressure. A highway can be regarded as a 1D road network, and the jammed clusters on the highway are, therefore, 2D (one of space and one of time). Indeed, as can be seen from Fig. 2 *E* and *F*, the distributions of cluster size and recovery duration of the 2D jammed clusters also show a clear scale-free scaling but with different typical exponents. As seen in *SI Appendix*, Fig. S6, the scaling exponents are also surprisingly stable and almost do not change from day to day. For the 1D highway, the scaling exponent for traffic resilience is much smaller than that of 2D city, suggesting higher chance of a larger jam and longer recovery duration. This lower resilience is probably due to the fact that, in jammed highways, no alternative routing paths are available for traffic flows, while jams in the city traffic network have more opportunities to be resolved. However, as shown in *SI Appendix*, Fig. S7, the resilience of urban traffic during the holiday is higher, with a higher exponent (2.69 ± 0.06) between October 1 and October 7 (the National Day of China) with significantly decreased total traffic demand.

**Fig. 4.**
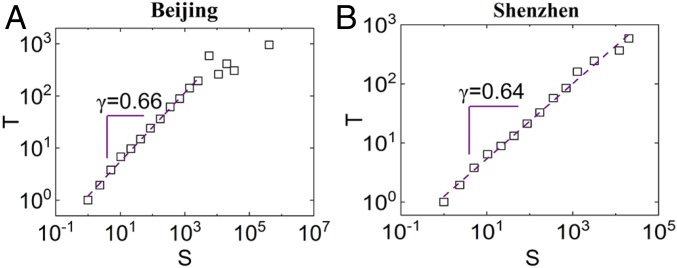
Recovery time vs. cluster size in (*A*) Beijing on October 26, 2015 and (*B*) Shenzhen on October 26, 2015.

To understand the relationship between traffic jam and recovery duration, we show in Fig. 4*A* that the recovery time of jammed clusters increases with cluster size with a scaling relation

$$T∼S^{\gamma },$$ [5]

where γ is the scaling exponent. This scaling exponent is found to be similar for both Beijing and Shenzhen. Moreover, this further indicates that the same general mechanism exists for all sizes of jams. For ecological and climate systems, it has been found that the recovery rate of the system bouncing back from perturbations becomes gradually slow when approaching the tipping point (44). While this is rarely observed and confirmed in engineering systems, transportation, as one of the largest complex engineering systems, is observed here to have the recovery duration growing with the increasing system failure size. We also test the relation between cluster size and recovery duration of jammed clusters on the Beijing–Shenyang Highway (*SI Appendix*, Fig. S8*C*) and find a different power law relation. The value of γ is also stable for all of the observed days as shown in *SI Appendix*, Fig. S8. Other than the temporal dimension of the spatiotemporal jammed clusters, we also tested the spatial dimension of the resilience clusters (*SI Appendix*, Fig. S9), and we found that the structures are self-affine and that the spatial dimension grows much slower than the temporal dimension.

Next, we ask if these three exponents α, β, and γ can be theoretically related. Indeed, if we assume that $P\left(S\right)∼S^{-\alpha }$, that $\Phi \left(T\right)∼T^{-\beta }$, and that $T∼S^{\gamma }$ (α, β, γ > 0), the exponents α, β, and γ should be related through the relation between the distributions (45),

$$P\left(S\right)=\Phi \left(T\right)\frac{dT}{dS},$$ [6]

from which we obtain

$$\gamma =\frac{\alpha -1}{\beta -1}.$$ [7]

Indeed, Eq. **7** is valid within the error bars found for these exponents [the comparison of actual value of γ with the theoretical value (Eq. **7**) of γ is in *SI Appendix*, Fig. S8 ].

## Discussion

In summary, we have developed an intuitive definition of traffic resilience based on the spatiotemporal evolution of jammed clusters. We find based on real data that both spatiotemporal cluster size of jams and their recovery duration follow a scale-free distribution, suggesting universal responses of transportation systems to different perturbation scenarios. Note that, on the temporal scale, ref. 46 discusses the scaling relation between the lifetime of the traffic jam and system size in a 1D lattice of the cellular automaton model. On the spatial scale, ref. 47 has also found spatial correlations in traffic flow fluctuations that show a power law decay. While their findings of scaling on the spatial or temporal scale are consistent with our results, we identify here a combined spatiotemporal scaling of traffic jams. This may help to design mitigation methods for viewing jams in a stereo way. For example, similar delay in different cities with different efficiencies (48) may be the result of similar scaling of spatiotemporal congestions formed by the self-organized criticality mechanism. These scaling relations are predictable and independent of fluctuating traffic demand on different days in two different cities. The current absence of a suitable definition of traffic resilience could have been the reason for the shortage of efficient allocation of mitigation resources and policy design for risk management. Our findings suggest that urban traffic in different cities could be classified into a few groups, with each group being characterized by the same scaling function and the same set of scaling exponents. Each group (with its intrinsic response to various perturbations) requires different resilience management. Our result is of great theoretical interest, motivating (in analogy to critical phenomena and the universality principle) theoretical studies regarding these intriguing questions: which traffic management variables are critical for determining the resilience scaling functions, and which are irrelevant?

Moreover, the indications found here in universality of traffic resilience are also of much practical interest. Specifically, when performing resilience management methods (49–51), one may pick the most tractable traffic jam to study, which will help to predict behaviors of all of the other jams in the same universality class, especially the likelihood of extreme event by statistical extrapolations (52). The relationship between the cluster size and recovery duration can be applied to predict (53) the congestion influence and the behavior of a certain jam size, which can help the decision-making process in the management of transportation. Meanwhile, additional studies, including model simulations, are needed to test and explain the universal characteristics of our results.

While many studies focus mainly on traffic control on the macroscopic or microscopic scale with dimensionless objectives, including travel time or speed (54), here we propose a resilience indicator in the combined spatiotemporal dimension. While it becomes increasingly difficult (if not impossible) to avoid traffic congestion, in this study, we wish to understand the development and recovery behavior of congestion. Our method may help to design traffic control methods to slow, diminish, and shrink the spatiotemporal jammed clusters, leading to improved system resilience. By plotting (*SI Appendix*, Fig. S4) day by day, for example, the maximal cross-section of the spatiotemporal congestion cluster in Beijing, this stable cross-section will first help to locate the high-frequency congestion region in real-time traffic. Existing traffic controls are aiming at signal control or road pricing to achieve the optimal operations. For urban traffic, these methods now focus on global flow properties, including macroscopic fundamental diagrams (55), and rarely consider the spatiotemporal organization of jam in the controlled region, which is the focus of this manuscript. Using our findings, design schemes and control methods could help to disintegrate the growth of jammed clusters and balance the spatial organization of traffic flow in a more accurate and controlled manner. Furthermore, the scaling laws that we identified can help to predict cluster jams above certain values and balance cost and efficiency. Future works should also focus, based on our approach, on evaluating the traffic resilience in other cities and other infrastructures when appropriate data become available. With the broad range of applications of network resilience, developing innovative interdisciplinary approaches based on big data to identify and understand the origin of the scaling laws (56–58) of system resilience is thus a big future challenge.

A key gradient to achieve the sustainability for a given system is to learn from past failures and enhance the system resilience. In this study, the resilience definition is mainly applied to the traffic congestion scenario as the major failure of transportation. For other natural and engineering systems, such as ecological damages or communication failures, the relevant resilience can be generalized based on our definition, considering the spatiotemporal features of system adaptation and recovery under perturbation. The knowledge of system adaptation and recovery can help to better evaluate the system risk, predict the size of the damage in the system or even collapse, and better mitigate against various perturbations.

## Materials and Methods

### Traffic Dataset.

The static road network in Beijing contains over 39,000 road segments (links) and 27,000 intersections (nodes), while the Shenzhen road network contains about 18,000 road segments (links) and 12,000 intersections (nodes). The dataset covers GPS velocity records of both cities for 30 d during October 2015 with resolution of 1 min; they are recorded through floating cars. The Beijing–Shenyang Highway includes 567 end-to-end road segments (links) along the Beijing to Shenyang direction and 562 end-to-end road segments (links) along the Shenyang to Beijing direction. The dataset covers GPS velocity records from October 1 to October 7, 2015 with resolution of 5 min.

### Definition of 3D Jammed Clusters.

A dynamical traffic network can be constructed based on road topology information and high-resolution traffic velocity data. Roads with real-time velocity $v_{i}$ below the corresponding threshold $p_{i}$ are regarded as congested (detailed thresholds for different roads are shown in *SI Appendix*, Table S1). Congested roads at each instant can form spatially connected clusters, which will evolve along the time. Considering the temporal evolution of spatially connected clusters, we can regard the jam in a city as a 3D spatiotemporal network cluster. Accordingly, a 3D (two of space and one of time) cluster can be constructed representing the same jam during its entire lifetime. Note that, when a jammed cluster splits into two or more subclusters at a certain instant, all links and nodes in the subclusters still belong to the same 3D cluster due to their temporal connection. Similarly, when two (or more) jammed clusters merge, they will be regarded as a single 3D cluster. Cluster lifetime is defined as the timespan between the formation of the cluster at $t_{0}$ and the dissolution of the cluster at $t_{1}$ (*T* = $t_{1}$ − $t_{0}$ + 1).

## Supplementary Material

Supplementary File
