# Supplementary material for: Scale-free resilience of real traffic jams
Source: Proc Natl Acad Sci U S A. 2019 Apr 12;116(18):8673–8. doi: 10.1073/pnas.1814982116 (PMC6500150; doi:10.1073/pnas.1814982116)
Supplement: Supplementary File [file pnas.1814982116.sapp.pdf]

PNAS
www.pnas.org

Supplementary Information for

Scale-free Resilience of Real Traffic Jams

Limiao Zhang, Guanwen Zeng, Daqing Li*, Hai-Jun Huang*, H. Eugene Stanley*, Shlomo Havlin

Daqing Li
Email: daqingl@buaa.edu.cn
Hai-Jun Huang
Email: haijunhuang@buaa.edu.cn
H. Eugene Stanley
Email: hes@bu.edu

This PDF file includes:

Materials and Methods
Figs. S1 to S10
Tables S1 to S2
References for SI

www.pnas.org/cgi/doi/10.1073/pnas.1814982116

Table S1. Velocity thresholds of different road levels

No.	Road Level	Velocity Threshold (km/h)
1	Intercity Highway	40
2	Urban Expressway	20
3	National Highway	12
4	Provincial Road	12
5	County Road	10
6	Township Road / Other	10

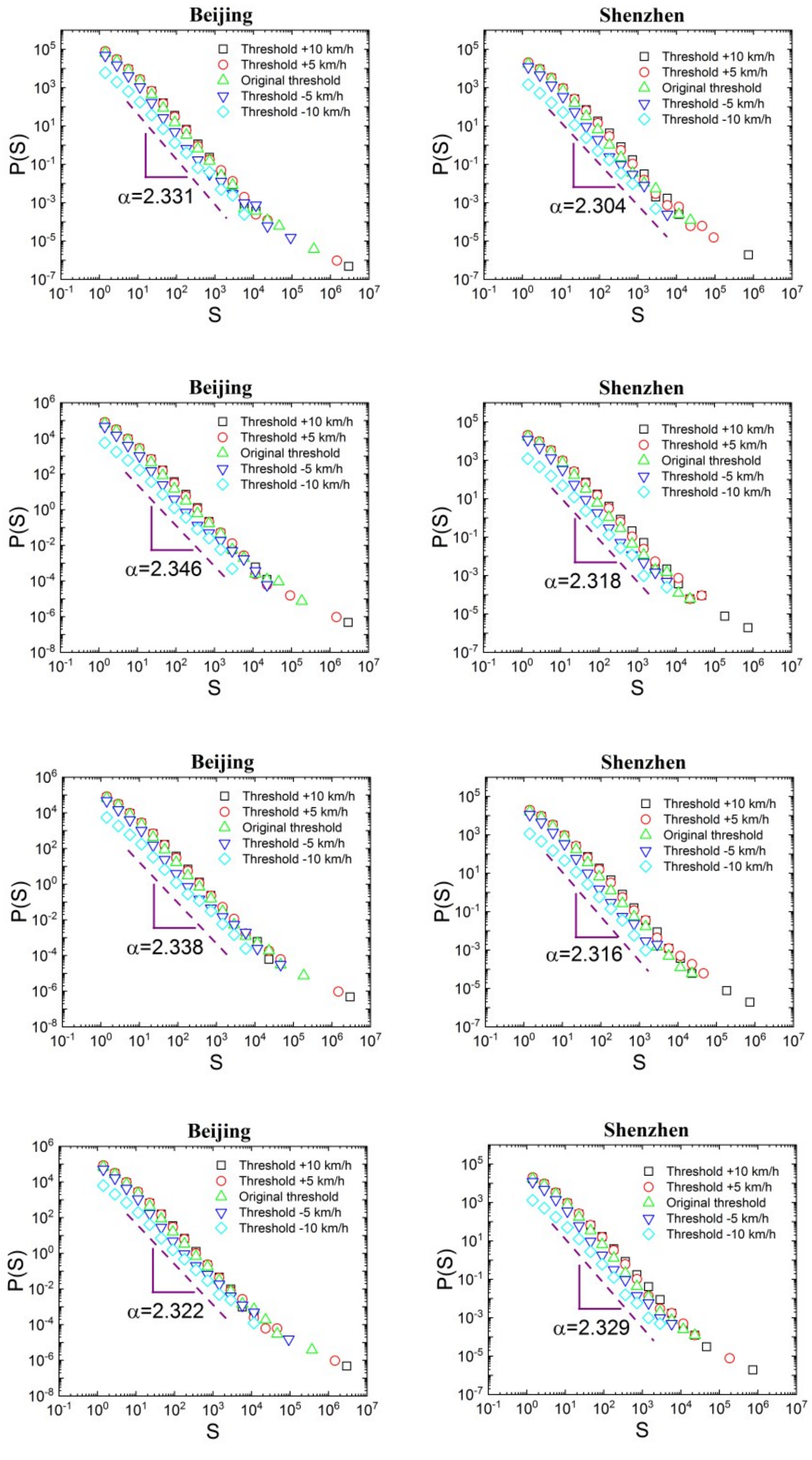

Fig. S1. Distribution of jammed cluster sizes in workdays from Oct. 26th to Oct. 29th (2015) in Beijing and Shenzhen using different speed thresholds. Dashed lines represent the fitting under the original official threshold.

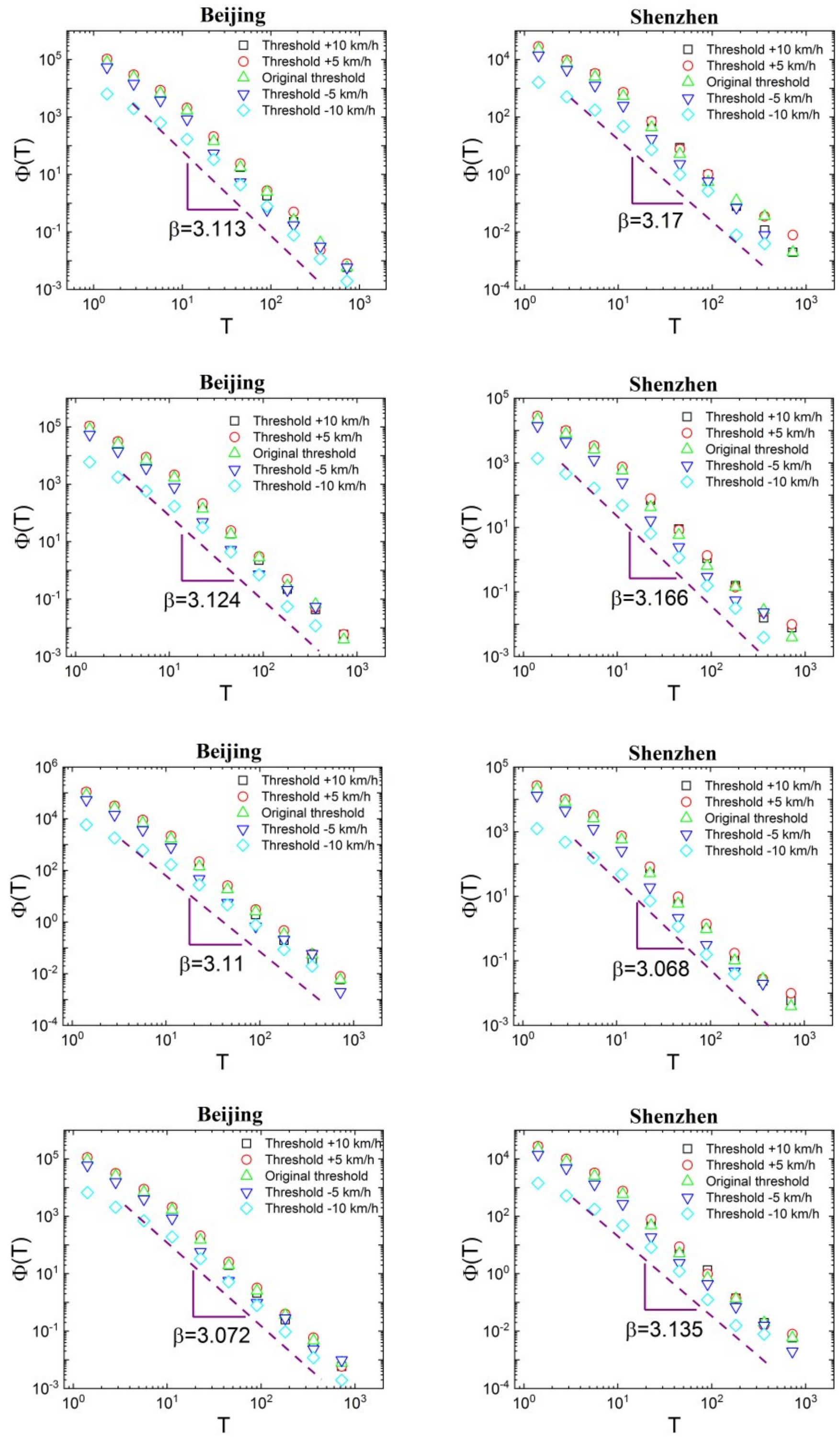

Fig. S2. Distribution of recovery durations in workdays from Oct. 26th to Oct. 29th (2015) in Beijing and Shenzhen using different speed thresholds. Dashed lines represent the fitting under the original official threshold.

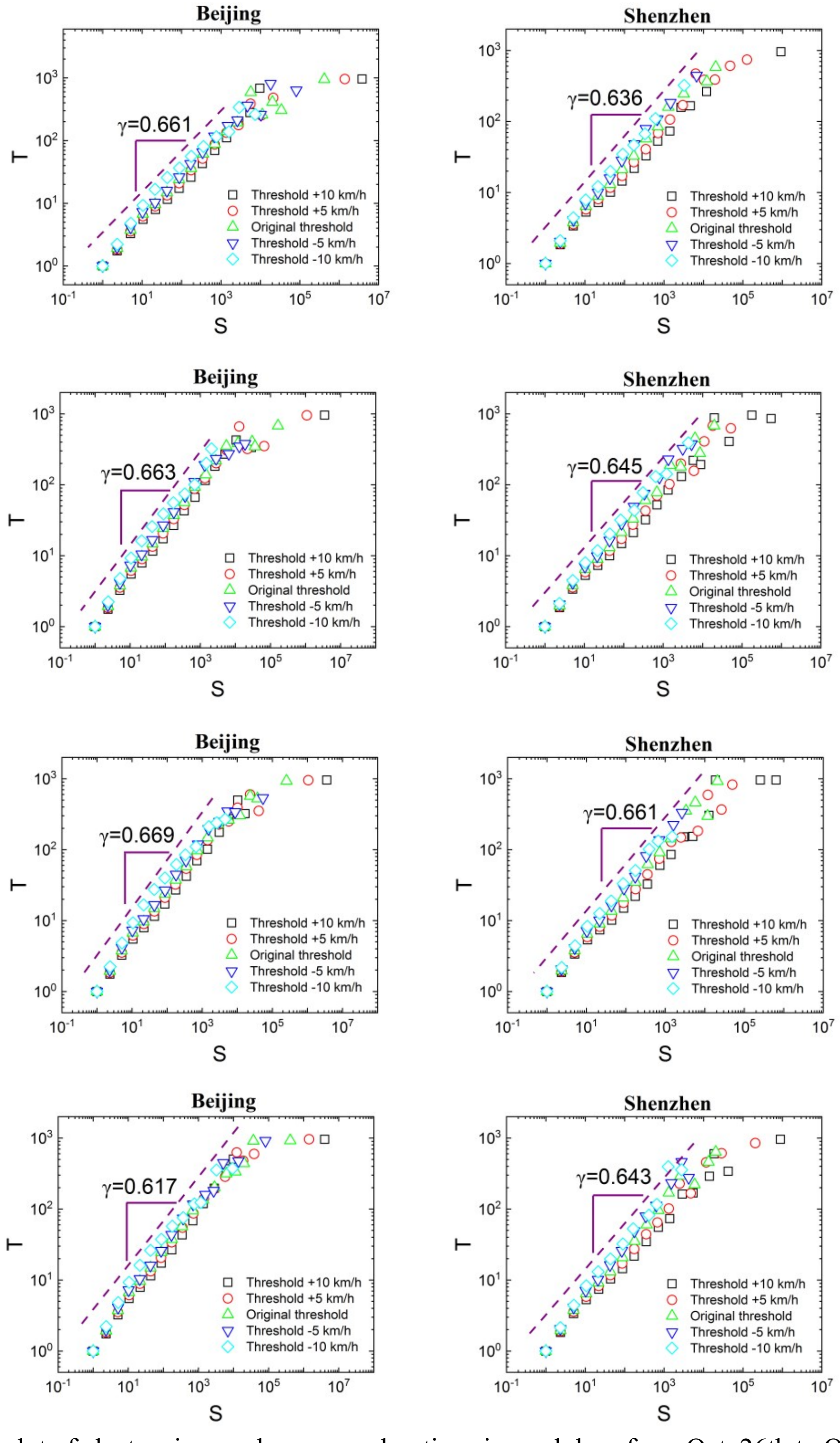

Fig. S3. Scatter plot of cluster sizes and recovery durations in workdays from Oct. 26th to Oct. 29th (2015) in Beijing and Shenzhen using different speed thresholds. Dashed lines represent the fitting under the original official threshold.

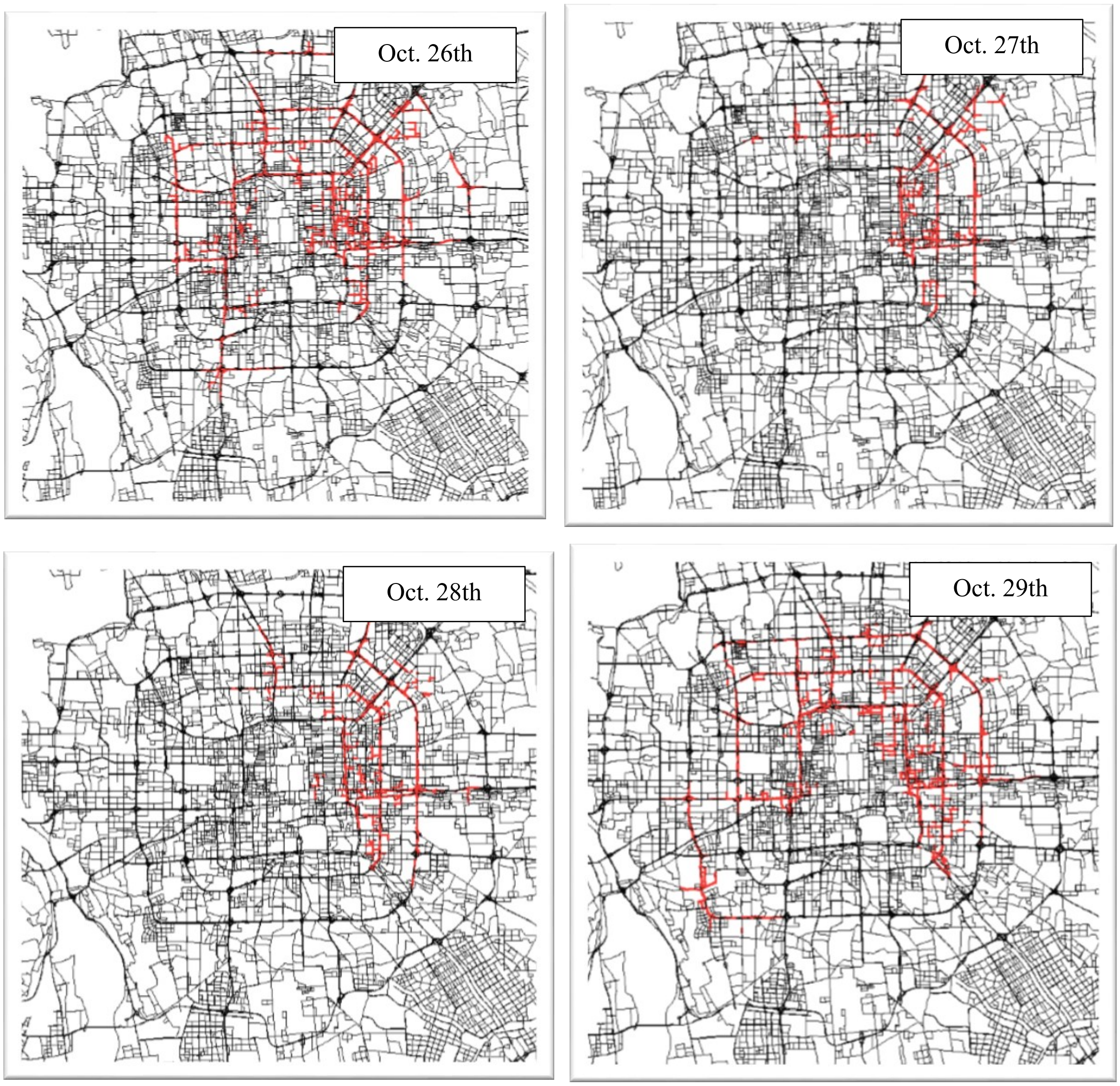

Fig. S4. Geographical locations of the maximal cross section jam, M_s^{max}, in four days between Oct. 26th and Oct. 29th (2015) in Beijing. The big overlap can be clearly seen.

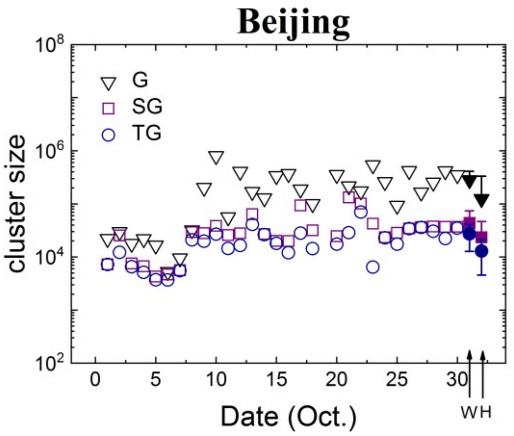

Fig. S5. The size of the first three largest jammed clusters as a function of date in Beijing. G: the largest cluster. SG: the second largest cluster. TG: the third largest cluster. The last two points (in the right) of each size represent the average jammed cluster sizes of workdays (W) and holidays (H), respectively. The largest jammed cluster sizes are obviously smaller in holidays (Oct. 1st-Oct. 7th 2015), due to less commuters compared to normal workdays.

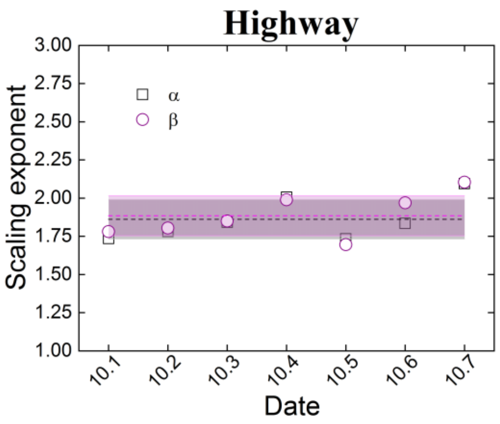

Fig. S6. Scaling exponents of the distributions of jammed cluster sizes and recovery times as a function of date of Beijing-Shenyang Highway. The shadow areas represent the error bars of α and β, separately.

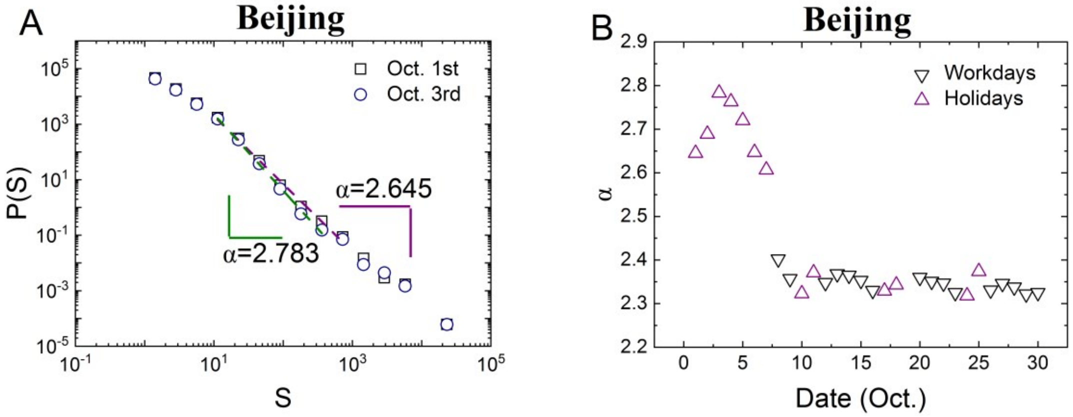

Fig. S7. (A) The distribution of jammed cluster sizes on typical holidays. (B) Scaling exponents of the distribution of jammed cluster sizes as a function of date in Beijing. The exponents in holidays Oct. 1st-Oct. 7th 2015 (2.69 ± 0.06) are clearly larger than those in workdays (2.35 ± 0.02), which indicates less large jammed clusters in holidays.

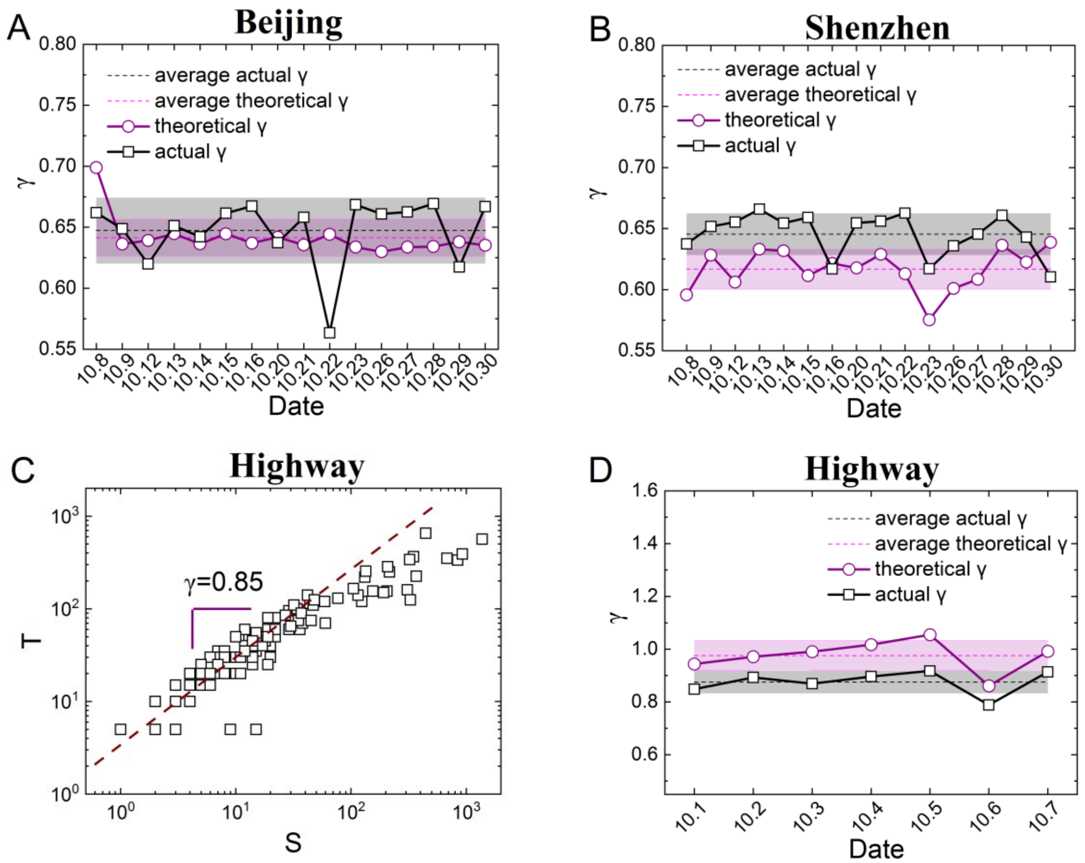

Fig. S8. The scaling exponents for recovery time versus jammed cluster size as a function of date: (A) for Beijing, (B) for Shenzhen. The shadow areas represent the error bars of γ. The theoretical values of γ are calculated using Eq. (7). (C) Typical scatter plots of recovery time versus cluster size of Beijing-Shenyang Highway on Oct. 1st 2015. (D) The scaling exponents of recovery time versus cluster size as a function of date of Beijing-Shenyang Highway.

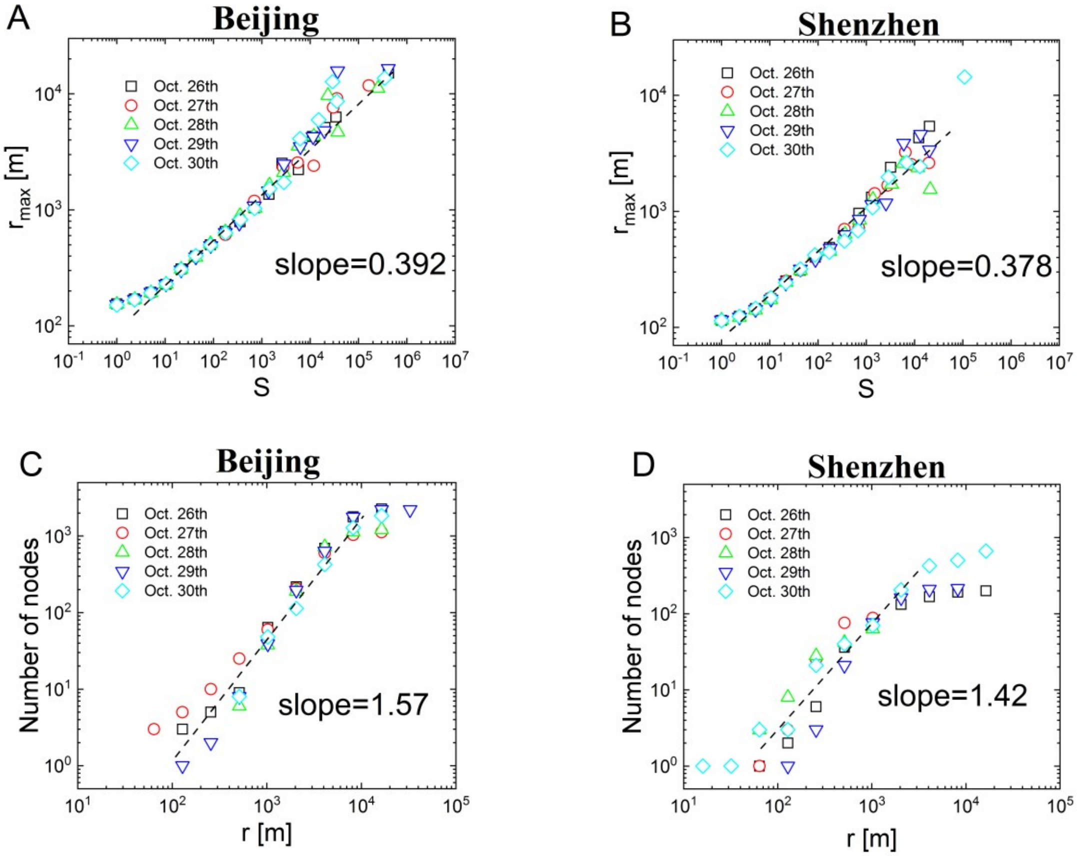

Fig. S9. Spatial dimension of the spatio-temporal clusters in both cities. The radius of the maximal cross section for each spatio-temporal cluster as a function of cluster size for (A) Beijing, (B) Shenzhen. The fractal dimension of the maximal cross sections for (C) Beijing, (D) Shenzhen.

Materials and Methods

Kolmogorov–Smirnov test (KS-test) for power law distribution hypothesis

We have applied the Kolmogorov–Smirnov test for our results to demonstrate that our findings of resilience and recover duration are indeed represented by power-law distributions (1, 2). We only concentrate on the data in a certain bounded range [a, b].

Following is our approach to test power-law hypothesis:

1) Define H_0: the data fits a power law distribution, and H_1: the data does not fit a power law distribution.

2) Estimate exponent α of the power-law scaling that best fits in the bounded binned data by MLEs (maximum likelihood estimates). The form of the estimated model is

$$P(x) = Cx^{-\alpha}, x\epsilon[a\ , b]. \tag{8}$$

Here we use the logarithmic binning scheme to determine the fitting area (2), which is determined by 3 parameters: the binning start x_{min} (which is in fact the a), the logarithmic base c, the number of binning intervals h. $b = x_{min} \times c^h$.

To get enough binning intervals, we chose $c = 2$ for all binning cases in our paper. $x_{\min}$ and h (also with $x_{\max}$ which can be directly derived from equation (8)) can be determined by the following process: for all combinations of $x_{\min}$ and h that fit well the power-law hypothesis (which means the p-value is greater than 0.1), the one contains more data (which calls for a rather small $x_{\min}$ and a rather large h) is selected.

The formalism of the *maximum likelihood function* (MLF) can be written as follows (3):

$$\log[L(\alpha)] = -n\log(x_{\min}^{1-\alpha} - x_{\max}^{1-\alpha}) + \sum_{i=1}^{h} n_i \log[(x_{\min}\cdot 2^{i-1})^{1-\alpha} - (x_{\min}\cdot 2^{i})^{1-\alpha}], \tag{9}$$

where n_i is the number of data points in bin i and $n = \sum_{i=1}^{h} n_i$ is the total amount of the fitted data.

This MLF approach has been generalized in reference (2). We solve the equation numerically to get the value of α for each combination of $x_{\min}$ and h, as shown in Table S2. The range for the fitting line in the scaling plots shows the range between the lower and upper cut-offs. For significance, p-values are also shown in Table S2 to indicate the goodness-of-fit (2).

3) Calculate the KS statistics, D, between the original data and the estimated model D is obtained by

$$D = \sup_{x}|F_{data}(x) - F_{model}(x)|, \tag{10}$$

where $F_{data}(x)$ and $F_{model}(x)$ are the cumulative distribution function of the real data and the estimated model, respectively.

4) Generate randomly n (here n = 1000) synthetic datasets of the same sample size from the fitted power-law model.

5) Calculate the KS statistic, D*, between each synthetic data and the model. Each D* is derived by

$$D^* = \sup_x \left|F_{syn}(x) - F_{model}(x)\right|, \tag{11}$$

where $F_{syn}(x)$ and $F_{model}(x)$ is the cumulative distribution function of the synthetic data and the estimated model, respectively.

6) Compare D with each D* and count the frequency that D is not larger than D*, denoted by p-value. If p-value is larger than or equal to 0.1, the hypothesis is regarded acceptable (1).

From Table S2 and Fig. S10, we conclude the assumption that the data fits power law distribution is acceptable.

Table S2. The results of KS test for power law distribution hypothesis

Table S2-1

KS Test for Size Distribution in Beijing				
**Date**	α	h	x_{min}	**p value**
10/8/2016	2.402	10	7	0.206
10/9/2016	2.357	10	8	0.416
10/12/2016	2.348	10	8	0.223
10/13/2016	2.368	10	10	0.177
10/14/2016	2.365	10	8	0.237
10/15/2016	2.353	10	8	0.425
10/16/2016	2.33	10	11	0.1
10/20/2016	2.36	10	10	0.285
10/21/2016	2.351	10	8	0.156
10/22/2016	2.347	10	10	0.16
10/23/2016	2.325	10	8	0.15
10/26/2016	2.331	10	8	0.47
10/27/2016	2.346	10	8	0.103
10/28/2016	2.338	10	8	0.933
10/29/2016	2.322	10	8	0.152
10/30/2016	2.325	10	8	0.609

Table S2-2

KS Test for Recovery Time Distribution in Beijing				
**Date**	α	h	x_{min}	**p value**
10/8/2016	3.006	8	15	0.181
10/9/2016	3.133	8	6	0.445
10/12/2016	3.109	8	6	0.177
10/13/2016	3.122	8	6	0.171
10/14/2016	3.145	8	6	0.257
10/15/2016	3.099	8	6	0.287
10/16/2016	3.088	8	6	0.457
10/20/2016	3.119	8	6	0.253
10/21/2016	3.126	8	6	0.291
10/22/2016	3.091	8	6	0.212
10/23/2016	3.091	8	6	0.963
10/26/2016	3.113	8	6	0.551
10/27/2016	3.124	8	6	0.321
10/28/2016	3.11	8	6	0.168
10/29/2016	3.072	8	6	0.463
10/30/2016	3.086	8	6	0.216

Table S2-3

KS Test for Size Distribution in Shenzhen				
**Date**	α	h	x_{min}	**p value**
10/8/2016	2.357	10	12	0.175
10/9/2016	2.362	10	15	0.226
10/12/2016	2.299	10	10	0.182
10/13/2016	2.356	10	16	0.111
10/14/2016	2.369	10	14	0.344
10/15/2016	2.351	10	13	0.228
10/16/2016	2.295	10	14	0.104
10/20/2016	2.333	10	12	0.371
10/21/2016	2.325	10	15	0.114
10/22/2016	2.33	10	11	0.112
10/23/2016	2.33	5	16	0.141
10/26/2016	2.304	10	10	0.163
10/27/2016	2.318	10	12	0.107
10/28/2016	2.316	10	15	0.219
10/29/2016	2.329	10	12	0.295
10/30/2016	2.31	10	16	0.182

Table S2-4

KS Test for Recovery Time Distribution in Shenzhen				
**Date**	α	h	x_{min}	**p value**
10/8/2016	3.278	8	6	0.686
10/9/2016	3.168	8	6	0.157
10/12/2016	3.143	8	6	0.275
10/13/2016	3.142	8	6	0.158
10/14/2016	3.167	8	6	0.34
10/15/2016	3.21	8	6	0.524
10/16/2016	3.084	8	6	0.423
10/20/2016	3.158	8	6	0.1
10/21/2016	3.107	8	6	0.297
10/22/2016	3.17	8	6	0.362
10/23/2016	3.312	8	7	0.214
10/26/2016	3.17	8	6	0.563
10/27/2016	3.166	8	6	0.247
10/28/2016	3.068	8	6	0.356
10/29/2016	3.135	8	6	0.1
10/30/2016	3.051	8	6	0.292

Table S2-5

KS Test for Size Distribution of Beijing-Shenyang Highway				
**Date**	α	h	x_{min}	**p value**
10/1/2016	1.736	10	1	0.1
10/2/2016	1.781	10	1	0.784
10/3/2016	1.842	10	1	0.968
10/4/2016	2.007	10	1	0.432
10/5/2016	1.733	10	1	0.562
10/6/2016	1.835	10	1	0.394
10/7/2016	2.095	10	1	0.856

Table S2-6

KS Test for Recovery Time Distribution of Beijing-Shenyang Highway				
**Date**	α	h	x_{min}	**p value**
10/1/2016	1.78	8	3	0.179
10/2/2016	1.804	8	3	0.3
10/3/2016	1.85	8	3	0.799
10/4/2016	1.99	8	3	0.518
10/5/2016	1.695	8	3	0.138
10/6/2016	1.97	8	3	0.155
10/7/2016	2.104	8	3	0.767

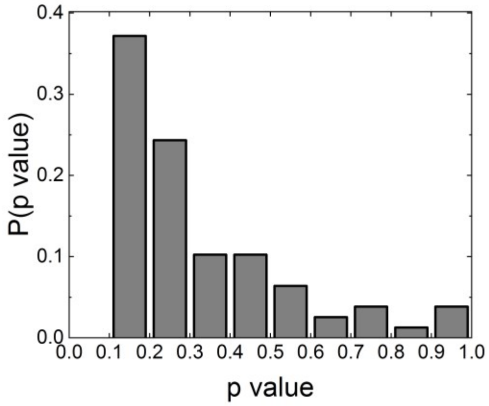

Fig. S10. The distribution of *p*-value in the KS test.

References

1. Clauset A, Shalizi CR, & Newman ME (2009) Power-law distributions in empirical data. *SIAM review* 51(4):661-703.

2. Virkar Y & Clauset A (2014) Power-law distributions in binned empirical data. *The Annals of Applied Statistics*:89-119.

3. Edwards AM, et al. (2007) Revisiting Lévy flight search patterns of wandering albatrosses, bumblebees and deer. *Nature* 449(7165):1044.
